# ACE-I induced angioedema: a case report and review of literature

**DOI:** 10.4076/1757-1626-2-7181

**Published:** 2009-07-27

**Authors:** Philip Babatunde Adebayo, Olutayo Christopher Alebiosu

**Affiliations:** 1 Department of Medicine, Olabisi Onabanjo University Teaching HospitalSagamu, Ogun StateNigeria; 2 Department of Medicine, Olabisi Onabanjo UniversityAgo-Iwoye, Ogun StateNigeria

## Abstract

**Introduction:**

Although rare, angioedema has been documented to occur following the administration of angiotensin-Converting Enzyme Inhibitors. Angiotensin-converting enzyme inhibitors are the leading cause of drug induced angioedema. Angiotensin-converting enzyme inhibitors induced angioedema is a class effect that can affect between 0.1% and 0.5% of patients taking the drug. It is rarely documented in Nigeria. Lisinopril is a commonly prescribed angiotensin-converting enzyme inhibitors-I which is considered to be generally safe and well tolerated. We report a case of angioedema following the use of lisinopril.

**Case presentation:**

A 52-year-old man, known hypertensive, presented with severe breathlessness on exertion, paroxysmal nocturnal dyspnoea and bilateral pedal swelling of six weeks duration. There was no history of allergy or atopy. He was managed with lisinopril 5 mg o.d, frusemide 40 mg daily, aspirin 150 mg daily and digoxin 0.25 mg daily.

He re-presented at the medical emergency unit of the hospital two days later with swellings involving the lips and the lower face of 10 hours duration. There was associated difficulty in swallowing but no stridor or hoarseness of voice. He did not have skin rashes or pruritus. There was no history of previous similar episodes.

Physical examination revealed a middle aged man with swollen lips and lower part of the face. The pharynx was oedematous. The respiratory and cardiovascular system examinations revealed features of hypertensive heart disease in biventricular failure. Clinical assessment of hypertensive heart disease in failure with Lisinopril induced Angioedema was made. The Naranjo probability scale indicated that this adverse drug event was probable.

Lisinopril was discontinued. After administration of corticosteroid and antihistamine, a complete resolution of the patient's symptoms was achieved. He was discharged to the medical outpatient unit of the hospital having recovered fully.

**Conclusion:**

This case is presented for the purposes of documentation since it is a rare occurrence among Nigerians.

## Introduction

Angioedema, which may be hereditary or non-hereditary, is an intense, usually disfiguring, temporary swelling of a localized body area involving the skin, mucosa and subcutaneous tissues. J. L. Milton first described angioedema in 1876 [1]. Quincke in 1882 [2] was the first to assign the name angioneurotic edema to the disease. The word ‘neurotic’ was used as part of the name in an attempt to describe the observed effect of mental stress on exacerbations of this disease. Areas commonly affected by angioedema include the face, lips, tongue, pharynx, the supraglottic area and, uncommonly, the subglottic area [3]. Angioedema may also involve the hands and feet, as well as the gastrointestinal mucous membranes and genitalia [3].

Hereditary angioedema is a rare autosomal dominant disorder, which is characterized by recurrent attacks of angioedema resulting from a deficiency of C1 esterase inhibitor enzyme [4]. The causes of non-hereditary angioedema are variable and include acquired C1 esterase inhibitor deficiency, which is a result of an auto-antibody to C1-INH, or generation of anti-idiotypic antibody to monoclonal immunoglobulins which occur in various B cell lymphoproliferative diseases and other malignancies [5]. Non-hereditary angioedema may also be idiopathic, or due to an allergic reaction to food, various inhalants, or immune complex diseases [5]. Angiotensin converting enzyme inhibitors now present as one of the most common causes of non-hereditary angioedema, accounting for 25-39% of cases [5]. Angioedema may be caused by other drugs as well, particularly aspirin and non-steroidal anti-inflammatory medications (NSAIDs), radio-contrast media, angiotensin II receptor antagonists, and certain antibiotics [5]. Several cases of severe angioedema have been reported following treatment with fibrinolytic agents [5] and a possible association with the use of estrogens, other antihypertensive drugs and psychotropic drugs has been suggested [5].

ACEI are used widely in the treatment of hypertension, heart failure, myocardial infarction, renal failure, and diabetic nephropathy. Over the last several years, the use of ACEI has increased enormously, and it is currently estimated that > 40 million people worldwide are receiving therapy with ACEI, which could lead to a greater prevalence of angioedema [6]. The relationship between drug intake and appearance of angioedema is extremely important in identification and subsequent withdrawal of the offending medication in drug induced type.

Soon after the development of ACEI, Wilkin et al reported angioedema and proposed enhanced kinin effects from inhibition of kininase II as the underlying mechanism [7]. ACEI have long been recognized to cause angioedema, with reported incidence varying from 0.1% to 1% [8]. Occurrence of angioedema has been reported with the use of all ACEI and it is considered a class-related side effect.

The incidence of ACEI-related angioedema is about 3 times higher in blacks than in white subjects; 4-fold higher incidence among patients with a history of drug rash; a 1.5-fold higher incidence in patients older than 65 years; an almost 2-fold higher incidence in patients with seasonal allergies. It also has a 14-fold higher risk of occurrence in the first week of therapy [9].

Inhibition of ACE blocks angiotensin conversion and reduces catabolism of bradykinin, a potent vasoactive peptide, which is degraded by ACE [10]. Hence, it has been speculated, and there is now some experimental evidence, that ACE inhibitors induce angioedema by increasing availability of bradykinin [11-13]. About 85% of cases of angioedema following exposure to ACEI resolve upon stopping the drug, it has been recommended that this simple measure, without further assessments, should be immediately taken in patients who experience angioedema while taking ACE inhibitors [14,15].

This case report is presented for the purposes of documentation since it is a rare occurrence among Nigerians.

## Case presentation

A 52-year-old Nigerian man of the Yoruba ethnic group, presented with severe breathlessness on exertion, paroxysmal nocturnal dyspnoea and bilateral pedal swelling of six weeks duration, having been referred from a private hospital. He was recently diagnosed hypertensive during consultation at the referring hospital, few weeks prior to presentation. He had smoked for 13 pack years and also consumed alcohol heavily (≥60 g/day) for thirty years. There was no history of food or drug allergy and no family history of atopy.

Physical examination revealed an acutely ill looking middle aged man. He was not pale. There was no periorbital or facial swelling. There was no significant peripheral lymphadenopathy. The main findings were in the cardiovascular system examination. The pulse was 106 beats per minute, regular and normal volume; the blood pressure was 145/100 mmHg supine. The apex beat was at the 6^th^ intercostals space, immediately lateral to the mid-clavicular line. Heart sounds SI, S2 and S3 gallop rhythm were heard. Chest examination showed few basal crepitations bilaterally. Fundoscopy revealed a grade 2 hypertensive retinopathy. All other systems were essentially normal. A clinical assessment of Hypertensive Heart Disease in failure was made. Although an in-patient management was offered, the patient however decline. He was managed on an out-patient basis; he was placed on lisinopril 5 mg daily, frusemide 40 mg daily, aspirin 150 mg daily and digoxin 0.25 mg daily.

The patient re-presented at the medical emergency unit of the hospital two days later with swollen lips and lower face of 10 hours duration. There was associated difficulty in swallowing but no stridor or hoarseness of his voice. There was no swelling of any other part of the body. He had neither skin rashes nor pruritus. There was no previous history of similar episode of swollen lips. Other aspects of his medical history did not show any abnormality and were not significant as to the likely cause of his disease state. Physical examination revealed a middle aged man with swollen lips and lower part of the face. The pharynx was also oedematous. He was not dyspnoeic.

The breath sounds were vesicular and there were no rales. The pulse rate was 94 beats per minute and respiratory rate was 20 breaths per minute respectively. His blood pressure was 140/90 mmHg. All other body system examination were essentially normal. A clinical assessment of lisinopril induced angioedema was made. The lisinopril was immediately discontinued. He was treated with intravenous hydrocortisone 200 mg stat and followed up with tabs prednisolone 10 mg tds and tabs chlorpheniramine 4 mg tds. He was seen at the outpatient unit two days later having recovered fully. The Naranjo probability scale indicated that this adverse drug event was probable.

## Discussion

Angioedema is the rapid swelling of the skin, mucosa and submucosa tissues [1]. It is a potentially life threatening condition. Most commonly, it occurs on the lips, tongue, face, hands or feet. Occasionally, the pharynx and larynx could be involved with patients presenting with respiratory embarrassment. The bowel and the penis could also be involved. The swelling is pale, non-itchy, but can be accompanied by urticaria. Symptoms last for one or two days.

The common form is mediated by allergy, (which could be to different substances like food, animal danders, extreme of temperatures, emotional stress and local trauma); the well defined inherited form known as hereditary angio-edema is due to deficiency and abnormal function of C1 esterase inhibitor.

ACEI are the leading cause of drug induced angioedema [8]. However, it has been reported as a side effect of some other medications including NSAIDS, Statins, Proton pump inhibitors, Selective serotonin reuptake inhibitor and other anti-depressant [3]. Although, the patient under discussion was also on aspirin during this period, it is not likely to have been responsible for his symptoms because his clinical improvement was sustained despite the fact that aspirin was not discontinued from his list of medications.

ACEI induced angioedema is a class effect that can affect between 0.1% and 0.5% of patients taking the drug. Although life threatening angioedema has a even lower frequency, fatal ACEI induced angioedema have been reported. Most episodes of angioedema reported occurred within the first week of therapy, but there are reports of angioedema as long as two years after the initiation of therapy [8]. Lisinopril which is more potent with a longer duration of action than either captopril or enalapril has enjoyed regular prescription from physicians and general practitioners. The frequency of angioedema associated with lisinopril is reported to be 0.1% [9].

ACEI induced angioedema is postulated to be due to reduced degradation of bradykinin. The kinins have multiple effects and are probably mediators of various cutaneous reactions including urticaria, flushing, pruritic reactions and angioedema. Angiotensin receptor blockers do not inhibit breakdown of bradykinins and were thought not to cause angioedema. However, there is now sufficient evidence to show an association with angioedema [9]. They may therefore not be a safe substitute in patients with prior ACE inhibitor induced angioedema.

ACEI associated angioedema may be accentuated in patients with renal impairment because of substantial drug accumulation in these patients. However, the patient did not have renal impairment. The primary management is to ensure an adequate airway. Endotracheal intubation and tracheostomy is required in severe cases with patient management in the intensive care unit. Corticosteroid, adrenaline and antihistamine are also useful.

This case is presented for the purpose of documentation since it is an unusual occurrence in this environment.

**Figure 1.**
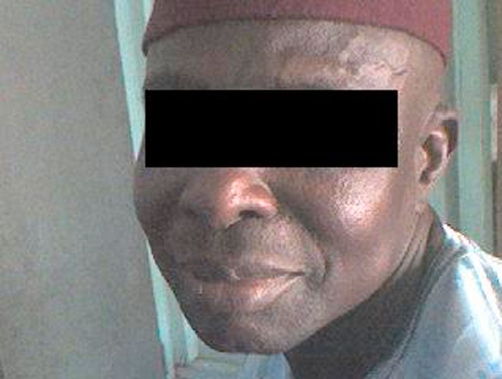


**Figure 2.**
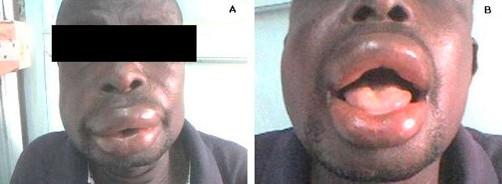


## Abbreviations

ACEI: Angiotensin-converting enzyme inhibitors
NSAIDs: Non-steroidal anti-inflammatory medications
